# Hematological and Thyroid Hormone Responses of Ethiopian Indigenous and Sasso Chickens Across Agroecologies: Implications for Climate Adaptation Under Smallholder Systems

**DOI:** 10.1155/vmi/2124011

**Published:** 2026-06-23

**Authors:** Chencha Chebo, Simret Betsha, Aberra Melesse

**Affiliations:** ^1^ Department of Animal Sciences, Arba Minch University, P.O. Box 21, Arba Minch, Ethiopia, amu.edu.et; ^2^ School of Animal and Range Sciences, Hawassa University, P.O. Box 5, Hawassa, Ethiopia, hu.edu.et

**Keywords:** adaptation physiology, agroecology, hematology, on-farm management, Sasso chickens

## Abstract

**Introduction:**

A bird’s production, physiological, immunological, and environmental adaptations can be evaluated through the hematological and biochemical profiles they possess. This study examined the adaptive characteristics of Ethiopian indigenous and Sasso chickens reared in rural farming systems based on hematological and hormonal parameters.

**Methods:**

Two hundred sixty‐two apparently healthy adult chickens, above one year, 122 indigenous and 140 Sasso breeds, sampled from three agroecologies, were examined for adaptive characteristics. The studied chickens were managed under farmer husbandry conditions without offering special care or treatment.

**Results:**

Most of the hematological profiles did not significantly (*p* > 0.05) differ due to breed effects; however, season and agroecology showed significant (*p* < 0.05) variations. The indigenous chickens had higher mean hematological values at all agroecologies and seasons; however, Sasso chickens had higher hematological profiles at highland and midland during wet seasons. Likewise, season and agroecology effects showed significant differences for triiodothyronine (T3) and thyroxine (T4) hormone levels. Sasso chickens exhibited higher mean T3 and T4 levels than indigenous chickens. The highland chickens, followed by the midlands, had higher T3 and T4 levels, and T3 levels were higher during the wet season, whereas T4 levels were higher during the dry season.

**Conclusion:**

The study provided useful insights, implying that season and agroecology had a significant effect on hematological and hormonal profiles and must be considered when distributing less‐adaptive commercial breeds to small‐scale farming systems. Further research involving molecular tools together with controlled thermal challenge experiments is suggested to ascertain the genetic potential for climatic resilience of the studied chickens.

## 1. Introduction

In recent years, there has been a remarkable rise in global environmental temperature, which has serious implications for the farming sector in tropical and subtropical regions of the world [1–3]. Changes in climatic conditions, production systems, and customer demand are also shifting the breeding goals for poultry from improving production traits to incorporating welfare, resilience, and disease resistance qualities [2, 4]. High ambient temperature often leads to stress‐associated problems, including production losses, metabolic changes, growth depression, and poor efficiency in birds [2, 5–7]. Heat stress disturbs the physiological status of birds, reducing feed intake and efficiency, which ultimately impacts performance and productivity, particularly in tropical and subtropical climates [8]. It is widely believed that the balance between the adaptive values of different gene types under varying environmental conditions plays a critical role in maintaining productivity [9, 10]. Furthermore, extreme weather events and increased volatility in weather patterns directly and indirectly impact chicken performance by altering feed and water availability [4, 11]. The future sustainable improvement of livestock production to meet the growing global demand for livestock products will therefore depend on our ability to utilize or develop climate‐resilient breeds [12].

Hematological profiles are key indicators for understanding the relationship between blood characteristics and environmental conditions [13] and can assist in selecting animals that are genetically resistant to certain diseases and environmental stress. Deviations in hematological profiles are routinely used to determine the impacts of environmental, nutritional, and pathological factors [14, 15]. Blood parameters such as white and red blood cell (RBC) counts, the packed cell volume (PCV%), the heterophil‐to‐lymphocyte (H/L) ratio, the hemoglobin (Hb) concentration, and red cell indices are key indicators of adaptive characteristics in animals under specific environmental conditions [16, 17]. These parameters are also indicative of breeding, feeding, and environmental and genetic factors that influence animal welfare and productivity [14]. Lymphocytes and heterophils are involved in adaptive and innate immune responses, respectively, and a high H/L ratio is a well‐known measure of physiological stress [2, 6, 18].

The hematological profile of chickens may vary with age, breed, sex, management system, and season [19, 20]. Other factors, such as stocking density, housing type, environmental conditions, and management practices, also influence hematological profiles in poultry [21]. Furthermore, the tolerance of chickens to a particular environmental stress can also be determined via the levels of thyroid hormones, such as triiodothyronine and thyroxine, in the blood [22, 23]. Thermal stress reduces thyroid hormone activity (T3 and T4), leading to a lower metabolic rate and reduced energy production [23, 24]. The same authors noted that T3 could be a reliable indicator of long‐term heat stress and could aid in selecting thermotolerant chicken breeds for hot climates. Thyroid hormones such as cortisol, T3, and T4 are crucial for thermogenesis, controlling metabolic heat production, and maintaining normal body temperature [1].

Given the rapid shifts in chicken production systems and emerging stressors due to climate change, continual assessment of the adaptability and productivity of chickens in tropical regions is essential. In the tropics, a massive importation and crossing of indigenous adaptive chickens with exotic breeds is becoming a common productivity improvement practice. However, due to uncontrolled breeding practices, there is no well‐designed strategy to monitor ideal exotic blood levels, which determines the adaptive status of chickens under on‐farm conditions. Thus, this study hypothesized that due to recognizable changes in climate, production, and management conditions across agroecologies, there might be significant adaptive variations of different chicken breeds under smallholder farming systems. Accordingly, this study investigated the adaptive potentials of indigenous and Sasso chickens reared in the Sidama region based on hematological and thyroid hormone profiles.

## 2. Materials and Methods

### 2.1. Description of the Study Areas

The study was conducted in three rural districts representing three agroecological zones of the Sidama regional state, Ethiopia. Districts were selected based on chicken production potential and agroecological distinctiveness. The first district, Shebedino, is located at 6°44′ to 6°84′ N and 37°92′ to 38° 60′ E, with an altitude range of 1146–1350 m above sea level, and is partly rooted in the Great Rift Valley. This district represents midland agroecology, with moderate temperatures ranging from 16°C to 25°C, and receives a mean annual rainfall of 1170 mm. Boricha district is the second district, which is situated between 6°46′N and 7°01′N longitude and 38°04′E and 38°24′E latitude. Boricha has mainly dry lowlands, which account for 78% of the total land area coverage and typically represent a lowland agroecology with warmer temperatures and a lower rainfall distribution. The third district is Hula, which typically represents highland agroecology and is characterized by cooler weather conditions with low temperatures and higher rainfall, with altitudes ranging from 2759 to 2829 m above sea level. The descriptions of the study districts were obtained from the Sidama Regional State Bureau of Agriculture and Livestock and Fishery Development for the 2023 fiscal year annual report (unpublished). In addition, the agroecological classifications of the study districts as highland, midland, and lowland were based on the guidelines of the Ethiopian Ministry of Agriculture [25].

### 2.2. Ethical Approval

We confirm that the ethical policies of the journal, as noted on the journal’s author guidelines page, have been adhered to, and an ethical review committee approval has been received from the Livestock and Fisheries Research Center (approval number AMU/LFRC/0236/23, dated 05/01/2023). All procedures were conducted in accordance with DIRECTIVE 2010/63/EU OF THE EUROPEAN PARLIAMENT AND OF THE COUNCIL of September 22, 2010, on the protection of animals used for scientific purposes, to ensure that the methods used do not cause the animals avoidable pain, suffering, distress, or lasting harm.

### 2.3. Study Chickens and Management

A total of 262 apparently healthy, above‐one‐year‐old chickens—122 indigenous and 140 Sasso—were sampled from three districts. Blood samples were collected in dry (January to February) and wet (July to August) seasons. Ninety households, 30 from each district, were purposefully selected based on their extensive experience in rearing indigenous and Sasso chickens for a period exceeding five years. All chickens were managed under farmer husbandry conditions without special care or treatment. Accordingly, indigenous chickens were fed on scavenging with a minimum feed supplementation of whole cereal grain and kitchen leftovers and were mainly housed within family dwellings. However, some farmers provided additional feed and constructed small, secure housing for Sasso chickens because of their better performance. Farmers own Sasso pullets and cockerels at the age of 45–49 days from private chick‐rearing farms, who receive day‐old chicks from Sasso chicken breeding and supplying companies (*EthioChicken*) located in various regions of the country. These chickens were administered scheduled vaccinations for common diseases at the main breeding company and rearing farms. On the other hand, indigenous chickens are owned by the breeding of farmers’ stock or are purchased from nearby local markets. There is no programmed vaccination practice for indigenous chickens in all study districts. Moreover, to minimize bias due to blood sample collection from many birds from the same flock, no more than 1 or 2 chickens were used for blood collection.

### 2.4. Field Blood Sample Collection and Processing

Blood samples were collected aseptically with a sterile syringe and needle from the wing vein of each bird by an experienced veterinarian. Before blood collection, the plumes were removed, and 70% ethanol alcohol was applied to distend the wing vein. A sterilized syringe and needle were used for each bird and then discarded immediately after use. Three milliliters of blood was collected from each bird, with 1 mL dispensed directly into labeled tubes containing the anticoagulant tri‐potassium ethylene diamine tetra‐acetic acid (K_3_EDTA) for hematological analysis. After being transferred into an EDTA tube, the tube was shaken gently 8–10 times to ensure proper mixing of the blood with the anticoagulant [26]. The tubes were then placed in an icebox filled with ice cubes to prevent sample deterioration. The remaining 2 mL of blood was transferred into serum separator tubes (SSTs) with a clot activator gel for total serum triiodothyronine and total thyroxine hormonal analyses. The SSTs were left at room temperature and allowed to coagulate for 30 min before being placed in an icebox. All the samples were transported to the Hawassa University Comprehensive Referral Hospital Laboratory within 8–10 h of collection for hematological and hormonal analysis.

For hematological analysis, due to the high viscosity of chicken blood, the prediluted procedure of an automated hematology analyzer (Mindray‐5130) was followed. A 20‐μL blood sample was mixed with 480 μL of aspirated diluent following a standard prediluted procedure. Then, the prediluted samples were analyzed for their hematological profile. The hematological parameters such as PCV%, RBC count (10^12^ cells/L), heterophil (%), lymphocyte (%), H/L ratio, Hb (g/dL), erythrocyte indices (mean corpuscular volume [MCV, fL]), mean corpuscular hemoglobin (MCH, pg/cell), and mean corpuscular hemoglobin concentration (MCHC, %) were examined. An auto‐hematology analyzer (Mindray‐5130) was used to analyze the targeted hematology parameters. Furthermore, blood samples collected in the SST were centrifuged at 4000 rpm for 5 min at 20°C, and the serum was stored at −80°C in Eppendorf test tubes until further analysis. Later, the total T3 and T4 hormones in the serum were measured via an automated chemistry analyzer (MAGLUMI 800, chemiluminescence immunoassay [CLIA] system).

### 2.5. Statistical Analysis

Before analysis, data were checked for normality and homogeneity of variance using quantile–quantile plots (Q–Q plots) and Bartlett’s test, respectively. Since the data were satisfying, the analyses of variance assumptions were computed by using the general linear model (GLM) procedures of the Statistical Analysis System [27] (Version 9.4) by fitting agroecology, season, sex, and breed as fixed effects. The differences between significant means were compared by Duncan’s multiple range test at the statistical significance level of *p* < 0.05. Mean and standard deviation values were computed for the examined hematological and hormonal profiles together with associated *p* values. The interaction effects between agroecology and season, as well as between breed and sex, were not of our interest; therefore, they were omitted from the statistical model. The following GLM model was applied.

(1) Yijkl=µ+Bi+Sj+Ck+Al+B∗Cik+B∗Ail+S∗Cjk+S∗Ajl+eijkl,

where *Y*_ijkl_ represents the response values for the hematology and hormone parameters, μ overall mean, *B*_*i*_ represents the *i*th breed effect (*i* = indigenous and Sasso), *S*_*j*_ represents the *j*th sex effect (*j* = male and female), *C*_*k*_ represents the *k*th season effect (*k* = wet and dry), *A*_*l*_ represents the *l*th agroecology effect (*l* = highland, midland, and lowland), (B ∗ C)_ik_, (B ∗ A)_il_, (S ∗ C)_ik_, and (S ∗ A)_jl_ refer to the interaction effects of *i*th breed, *j*th sex, *k*th season, and *l*th agroecology, and *e*_ijkl_ represents residual error.

## 3. Results

### 3.1. Hematological Profiles

Tables 1 and 2 present the main effects of breed, sex, agroecology, and season on the hematological profiles. Most of the hematological parameters significantly (*p* < 0.05) varied for agroecological and seasonal effects. However, breed and sex did not significantly (*p* > 0.05) affected the studied hematological profiles (Table 1). The overall mean values of the parameters were found to be within a physiologically acceptable range with justifiable deviations. Compared with Sasso chickens, indigenous chickens showed relatively higher, though not statistically significant, values for Hb concentration, PCV, MCV, and MCH values (Table 1). On the other hand, Sasso chickens had slightly higher values for lymphocytes, H:L ratio, and RBC counts. In terms of sex effect, male chickens exhibited significantly (*p* < 0.05) higher values than females for most of the parameters, with the exception of heterophils and MCHC, where no significant (*p* > 0.05) differences were detected.

**TABLE 1:** The main effect (means ± SD) of breed and sex on the hematological profiles.

Hematological profiles	Breed		Sex		Overall mean	Breed effect	Sex effect
	Indigenous	Sasso	Female	Male		*p* values	*p* values
Heterophils (%)	25.9 ± 6.2	27.9 ± 7.3	26.8 ± 6.5	25.9 ± 7.1	26.4 ± 6.8	0.328	0.264
Lymphocytes (%)	70.9 ± 5.3	71.3 ± 6.2	70.8 ± 5.4	71.5 ± 6.1	71.1 ± 5.7	0.637	0.330
H:L	0.34 ± 0.11	0.38 ± 0.11	0.36 ± 0.10	0.40 ± 0.37	0.37 ± 0.10	0.352	0.310
RBC (× 10^12^ cells/L)	3.32 ± 0.57	3.10 ± 0.70	3.08 ± 0.61	3.15 ± 0.67	3.11 ± 0.64	0.740	0.376
Hemoglobin conc. (g/dL)	16.5 ± 2.7	14.3 ± 3.1	16.4 ± 2.6	17.5 ± 3.3	16.4 ± 2.9	0.471	0.745
PCV (%)	42.6 ± 8.6	41.6 ± 10.0	41.8 ± 8.7	43.0 ± 10.1	42.0 ± 9.4	0.388	0.095
MCV (fL)	137 ± 10.1^a^	135 ± 9.1^b^	134 ± 8.9^b^	138 ± 9.8^a^	136 ± 9.6	0.015	< 0.001
MCH (pg)	60.4 ± 3.3	59.8 ± 3.1	59.8 ± 2.6	60.5 ± 3.7	60.1 ± 3.2	0.121	0.064
MCHC (g/dL)	44.1 ± 3.8	44.4 ± 3.8	44.8 ± 3.7	43.8 ± 3.9	44.3 ± 3.8	0.321	< 0.061

*Note:* ^a,b^Means in the same row with different superscripts are significantly different at *α* = 0.05. H:L refers to heterophil‐to‐lymphocyte ratio; conc. refers to concentration.

Abbreviations: MCH, mean corpuscular hemoglobin; MCHC, mean corpuscular hemoglobin concentration; MCV, mean corpuscular volume; PCV, packed cell volume; RBC, red blood cells; SD, standard deviation.

**TABLE 2:** The main effect (means ± SD) of agroecology and season on the hematological profiles.

Hematological profiles	Agroecology			Season		Overall mean	AEZ	Season
	Highland	Midland	Lowland	Dry	Wet		*p* values	*p* values
Heterophils (%)	28.2 ± 6.5^a^	26.1 ± 5.6^ab^	24.8 ± 7.3^b^	24.9 ± 6.0^b^	27.9 ± 7.2^a^	26.4 ± 6.8	0.002	< 0.001
Lymphocytes (%)	70.4 ± 5.9	71.1 ± 4.8	71.9 ± 6.1	71.1 ± 5.4	71.2 ± 6.1	71.1 ± 5.7	0.120	0.851
H:L	0.40 ± 0.11^a^	0.37 ± 0.08^ab^	0.35 ± 0.11^b^	0.35 ± 0.09^b^	0.39 ± 0.1^a^	0.37 ± 0.10	< 0.001	< 0.001
RBC (x10^12^ cells/L)	3.43 ± 0.58^a^	3.10 ± 0.67^b^	2.85 ± 0.53^c^	3.05 ± 0.65	3.2 ± 0.61	3.11 ± 0.64	< 0.001	0.118
Hemoglobin conc. (g/dL)	17.1 ± 2.6^a^	16.12 ± 2.8^b^	15.9 ± 3.2^c^	16.1 ± 3.2	16.7 ± 2.7	16.4 ± 2.9	0.015	0.102
PCV, %	45.3 ± 9.4^a^	39.3 ± 9.7^c^	41.9 ± 8.2^b^	44.9 ± 8.9^a^	39.3 ± 8.3^b^	42.0 ± 9.4	< 0.001	< 0.001
MCV (fL)	135 ± 9.5^b^	139 ± 9.7^a^	133 ± 7.3^c^	131 ± 9.4^b^	141 ± 6.8^a^	136 ± 9.6	< 0.001	< 0.001
MCH (pg)	61.3 ± 2.3^a^	59.8 ± 3.2^b^	57.1 ± 3.6^b^	60.6 ± 3.5	59.8 ± 2.8	60.1 ± 3.2	< 0.001	0.147
MCHC (g/dL)	47.3 ± 3.1^a^	45.2 ± 4.4^a^	42.6 ± 3.3^b^	46.3 ± 4.1^b^	42.2 ± 3.9^b^	44.3 ± 3.8	< 0.001	< 0.001

*Note:* ^a,b,ab,c^Means in the same row with different superscripts are significantly different at *α* = 0.05; AEZ refers to the agroecological zone; H:L refers to heterophil‐to‐lymphocyte ratio; conc. refers to concentration.

Abbreviations: MCH, mean corpuscular hemoglobin; MCHC, mean corpuscular hemoglobin concentration; MCV, mean corpuscular volume; PCV, packed cell volume; RBC, red blood cells; SD, standard deviation.

As shown in Table 2, significant (*p* < 0.05) variation was observed for all hematological parameters across agroecologies, with the exception of lymphocytes. Season had shown a significant (*p* < 0.05) effect on the level of heterophils, PCV%, MCV, and MCHC. Concerning the agroecology, highland chickens had significantly higher heterophils, H:L ratio, RBC, Hb concentration, and MCH values, followed by midland chickens. In contrast, midland chickens had the highest PCV, whereas lowland chickens exhibited higher MCV and lymphocytes. Season influenced the hematological profiles, with significantly higher MCH and MCHC values measured during the dry season. In contrast, the wet season was associated with significantly greater heterophil, H:L ratios, RBC counts, Hb levels, percentages of PCV, and MCV values.

For most of the parameters, the interactions among sex, breed, agroecology, and season did not show significant (*p* > 0.05) variations (Tables 3 and 4). However, PCV, MCV, MCH, and MCHC values were significant (*p* < 0.05) for breed and agroecology interaction, whereas only MCHC was found significant (*p* = 0.027) for breed and season interaction. In all agroecologies, for most of the hematological profiles, indigenous chickens had higher mean values; however, Sasso chickens at highland and midland agroecologies had higher mean values than at lowland. Likewise, during wet seasons, higher mean values were obtained than in the dry seasons for Sasso chickens, except for a few parameters. However, indigenous chickens had higher mean values than Sasso chickens during both dry and wet seasons.

**TABLE 3:** Interaction effects (means) of breed, season, and agroecology on hematological profiles.

Hematological profiles	Agroecology						Season				Breed ∗ AEZ	Breed ∗ season
Breed	Highland		Midland		Lowland		Dry		Wet		*p* values	*p* values
	Indi.	Sasso	Indi.	Sasso	Indi.	Sasso	Indi.	Sasso	Indi.	Sasso		
Heterophils (%)	27.5	28.8	26.7	25.4	23.6	26.0	24.0	25.8	27.9	27.7	0.128	0.144
Lymphocyte (%)	70.6	70.2	70.6	71.5	71.4	72.2	71.3	70.7	70.5	72.0	0.582	0.120
H:L	0.40	0.42	0.38	0.36	0.33	0.37	0.33	0.37	0.40	0.39	0.091	0.082
RBC (× 10^12^ cells/L)	3.2	3.0	3.3	3.5	2.8	2.9	3.0	3.0	3.1	3.2	0.085	0.837
Hemoglobin conc. (g/dL)	17.8	16.5	15.7	16.3	16.2	16.1	16.5	15.9	16.6	16.8	0.062	0.191
PCV (%)	44.3^a^	40.4^b^	43.9^a^	46.7	39.5	39.2	39.8	39.4	45.3	44.8	0.016	0.904
MCV (fL)	139.1^a^	133.4^b^	133.5^a^	132.9^b^	139.0	139.2	131.2	130.2	143.2	140.2	0.003	0.208
MCH (pg)	62.1^a^	60.7^b^	59.5^b^	60.2^a^	59.8^a^	58.6^b^	61.1	59.9	59.8	59.7	0.036	0.144
MCHC (g/dL)	44.8^b^	45.7^a^	44.8^b^	45.5^a^	43.2^a^	42.1^b^	46.7	46.3	41.8^b^	42.6^a^	0.009	0.027

*Note:* ^a,b^Means in the same row with different superscripts are significantly different at *α* = 0.05; AEZ refers to the agroecological zone; H:L refers to heterophil‐to‐lymphocyte ratio; conc. refers to concentration; Indi. refers to indigenous chickens.

Abbreviations: MCH, mean corpuscular hemoglobin; MCHC, mean corpuscular hemoglobin concentration; MCV, mean corpuscular volume; PCV, packed cell volume; RBC, red blood cells; SD, standard deviation.

**TABLE 4:** Interaction effects (means) of sex, season, and agroecology on hematological profiles.

Hematological profiles	Agroecology						Season				Sex ∗ AEZ	Sex ∗ season
	Highland		Midland		Lowland		Dry		Wet		*p* value	*p* values
Sex effect	M	F	M	F	M	F	M	F	M	F		
Heterophils (%)	26.5	29.6	26.7	25.5	24.6	25.0	24.3	25.5	27.6	27.9	0.088	0.548
Lymphocytes (%)	71.2	69.8	70.3	71.8	72.7	71.0	71.4	70.7	71.5	71.0	0.157	0.848
H:L	0.38	0.43	0.38	0.358	0.35	0.43	0.34	0.36	0.40	0.39	0.051	0.455
RBC (× 10^12^ cells/L)	3.33^a^	2.94^b^	3.26^a^	3.55	2.90	2.81	3.1	3.0	3.2	3.2	< 0.001	0.473
Hemoglobin conc. (g/dL)	17.8	‘16.9	15.6	16.3	16.1	16.2	16.2	16.3	16.8	16.5	0.167	0.573
PCV (%)	45.5^a^	39.8^b^	43.8^b^	46.7^a^	40.6^a^	38.2^b^	40.8	38.6	45.8	44.5	0.005	0.702
MCV (fL)	138.1	134.9	135.5	131.6	141.5	136.7	132.8	129.2	143.8	139.6	0.728	0.709
MCH (pg)	62.3	60.8	60.2	59.7	59.6	58.8	61.0	60.2	60.3	59.2	0.442	0.716
MCHC (g/dL)	45.3	45.2	44.7	45.6	42.2	43.1	46.2	46.8	42.0	42.5	0.303	0.831

*Note:* ^a,b^Means in the same row with different superscripts are significantly different at *α* = 0.05; AEZ refers to the agroecological zone; H:L refers to heterophil‐to‐lymphocyte ratio; conc. refers to concentration; M refers to male; F refers to female.

Abbreviations: MCH, mean corpuscular hemoglobin; MCHC, mean corpuscular hemoglobin concentration; MCV, mean corpuscular volume; PCV, packed cell volume; RBC, red blood cells.

### 3.2. Thyroid Hormone Profiles

All fixed effects, except breed, were found to have a significant (*p* < 0.05) effect on the levels of the T3 and T4 hormones (Table 5). Compared with female chickens, male chickens showed higher concentrations of both T3 and T4. Regarding the agroecology, highland chickens had the highest T3 concentration, followed by the midland chickens, whereas the midland chickens had the highest T4 concentration. During the dry season, higher T4 and lower T3 concentrations were obtained. Although not statistically significant (*p* > 0.05), Sasso chickens had slightly higher T4 concentrations than the indigenous chickens, whereas significantly (*p* < 0.05) higher T3 levels were detected in Sasso chickens. In addition, significant (*p* < 0.001) interaction effects were detected between season and sex and between season and agroecology at the T3 level. For T4 levels, significant (*p* < 0.05) interaction effects were observed between season and agroecology and breed. However, the most interesting interaction effects among breed, season, and agroecology were not found to be significant (*p* > 0.05), except for breed interaction with season for T4 concentration (*p* = 0.021).

**TABLE 5:** The main and interaction effects of sex, breed, agroecology, and season on total T3 and T4 concentrations.

Effects		Hormone concentration (means ± SD)	
		Total tri‐idothyronine (T3, nmol/L)	Total thyroxine (T4, nmol/L)
Sex	M	3.8 ± 0.90^a^	12.1 ± 4.8^a^
	F	3.4 ± 0.81^b^	9.8 ± 4.7^b^

Breed	Indigenous	3.6 ± 0.92	10.2 ± 4.4
	Sasso breed	3.7 ± 0.91	11.4 ± 5.1

Agroecology	Highland	3.9 ± 0.90^a^	11.1 ± 5.2^a^
	Midland	3.7 ± 0.81^ab^	11.9 ± 4.5^a^
	Lowland	3.3 ± 0.82^b^	9.7 ± 4.6^b^

Season	Dry	3.5 ± 0.91^b^	12.0 ± 4.4^a^
	Wet	3.8 ± 0.92^a^	9.9 ± 5.1^b^

Overall mean		3.62 ± 0.92	10.93 ± 4.85

**Main effects**		**p** **values**	**p** **values**

Sex		< 0.001	< 0.001
Breed		0.149	0.060
Agroecology		< 0.001	0.009
Season		0.004	< 0.001

**Interaction effects**			

Breed ∗ sex		0.811	0.259
Agroecology ∗ sex		0.056	0.354
Sex ∗ season		< 0.001	0.285
Breed ∗ agroecology		0.448	0.926
Breed ∗ season		0.595	0.021
Agroecology ∗ season		< 0.001	0.014

*Note:* ^a,b,ab^Means in the same column with different superscripts are significantly different at *α* = 0.05; M refers to male; F refers to female.

Abbreviation: SD, standard deviation.

## 4. Discussion

### 4.1. Hematological Profiles

#### 4.1.1. Breed Effect

The lack of significant (*p* > 0.05) differences between indigenous and Sasso chickens for most of the hematological parameters might be due to the good adaptive merit of Sasso chickens to various production and management conditions in the tropics. Sasso chickens are dual types and known for their heat tolerance and good foraging ability, which allows them to perform better in different regions of the tropics [28]. The slightly higher Hb concentration and RBC count observed in indigenous chickens compared to Sasso chickens suggested that different breeds may have different Hb concentrations for oxygen utilization (Subhadarsini and Silpa [20] and Muneer et al. [29]). Bora et al. [30] suggested that the higher Hb levels in indigenous chickens may be due to their increased activity levels compared with the exotic breeds under scavenging conditions. Studies have also shown that RBCs serve as transporters of O_2_ and CO_2_ through Hb [31]; thus, a lower number of RBCs possesses a lower concentration of Hb, which in turn leads to a reduced amount of O_2_ being transported to the tissue and the CO_2_ level being returned to the lungs [32].

The percent of PCV%) of the indigenous chickens in this study (42.6%) falls within the range of values (42.4%–52.6%) reported by Panigrahy [33] for Vanaraja indigenous chickens and the 35%–48% range for indigenous chickens in Iran reported by Bahman et al. [34] and Abdi‐Hachesoo et al. [35]. Similarly, Bora et al. [30] reported the mean values for PCV for Rajasri (45.5%), Kadaknath (43.6%), and Aseel chickens (41.8%) in India, which aligns with our findings. The PCV values reported by Muneer et al. [29] ranged from 31.5% to 42.2% for broilers and indigenous chickens, respectively. Abdulazeez et al. [36] reported lower PCV (32.9%), Hb (11.8 g/dL), RBC (2.3 × 10^12^ cells/L), and MCHC (35.8%) values for broilers than those reported in the present study. On the other hand, Adegoke et al. [37] reported much lower PCV (30%), RBC (2.6 × 10^12^ cells/L), MCH (37.9 pg), MCHC (33.2 g/dL), and MCV (11.37 fL) values for Arbor Acres broiler chickens than those reported in the present study, indicating that the genetics of chickens had contributed to the observed difference.

The observed MCV values for indigenous (137 fL) and Sasso (135 fL) chickens were consistent with the findings of Muneer et al. [29], who reported MCV values of 118.7 fL for indigenous chickens and 126.7 fL for broilers. However, these authors reported MCH values of 31.7 pg for broilers and 32.3 pg for indigenous chickens, as well as MCHC values of 25 and 27.2 g/dL for broilers and indigenous chickens, respectively, which were lower than the values recorded in the current study. Payvastegan et al. [38] reported an Hb concentration of 10.92 g/dL, an RBC count of 3.3 × 10^12^ cells/L, and a PCV of 30.8% for Ross 308 finisher broilers, which aligns with the current results for Sasso chickens. The variations observed between indigenous and broiler chickens for different hematological profiles were due to the genetic differences developed through various management, breeding, and selection strategies over time.

#### 4.1.2. Sex Effect

The higher and statistically significant (*p* < 0.05) mean values observed in male chickens are consistent with findings from several authors. For instance, Adewole et al. [39] reported that sex had significant effects on the hematological and serum biochemical indices of tropical indigenous chickens, with males having higher values. Similarly, the PCV, RBC, and Hb values were significantly (*p* < 0.05) higher in male Vanaraja chickens than in female chickens [33]. Addass et al. [40] also reported a significant sex effect, with males showing higher PCV and RBC profiles. The same authors reported that most hematological parameters in male indigenous chickens were higher than those in females. Similarly, Simaraks et al. [41] reported that male chickens had higher Hb and PCV values than females. Consistently, several authors [33, 40] reported higher PCV values in males. Abdi‐Hachesoo et al. [35] justified that the higher values observed in males might be due to the effect of the androgen hormone.

Furthermore, the observed H:L ratios ranged from 0.36 to 0.40, which were in agreement with the findings of Adewole et al. [39], with values of 0.3 for females and 0.4 for males for indigenous chickens of the FUNAAB Alpha and Yoruba ecotypes of Nigeria. Similarly, Panigrahy et al. [33] reported that the H:L ratio ranged from 0.38 to 0.4 for male and female Vanaraja chickens in India, which was consistent with our findings. Studies suggest that the H:L ratio remains relatively stable over the long term, with its high heritability supporting its use as an indicator of disease resistance in poultry breeding [42].

#### 4.1.3. Season Effect

A higher (not statistically significant, *p* > 0.05) RBC count was observed during the wet season than during the dry season, which aligns with findings from Pandian et al. [43] in adult Leghorn chickens and Mohamed et al. [44] in Ross and Cobb broilers. Similarly, Panigrahy et al. [33] reported high Hb concentrations during dry seasons due to heat stress, suggesting that increased Hb levels may be an adaptive response to meet the energy demands required for survival under stressful conditions.

The higher PCV during the dry season in this study may be attributed to hemoconcentration caused by dehydration due to high temperature, particularly in lowlands. This might also be due to increased PCV, which may be related to the increased metabolic activity required to meet the increased energy demands for maintenance and growth during scavenging conditions [45]. Previous studies have reported that environmental stress leads to decreased hematocrit values [46, 47], and this decline in hematocrit levels is often associated with feed and water shortages during dry seasons, as observed in lowland areas of the Sidama region in the present study. Moreover, Badruzzaman et al. [48] reported that high temperatures in tropical countries, particularly during the dry season, contribute to the prevalence of infectious and contagious poultry diseases such as Newcastle disease and Gumboro sickness, which can change the normal levels of blood parameters.

Furthermore, the low levels of MCH, MCV, and MCHC observed during the dry season in the present study align with findings in Sudanese chicken ecotypes [49], Ross and Cobb strains [44], Leghorn chickens [43], and native Vanaraja chickens of India [33]. The authors suggested that the reduction in this parameter may indicate a decrease in the Hb concentration during the dry season. In addition, a decreased MCV was also observed, which might be due to a higher RBC count during the dry season. Similar findings were reported in Ross and Cobb strains by Mohamed et al. [44]. Several studies have consistently reported increased H:L ratios in avian species under heat stress during hot climatic or induced stress conditions [47, 50, 51]. In contrast to these findings, Panigrahy et al. [33] reported lower H:L ratios during the dry season (0.38–0.39) than during the wet season (0.4–0.43) in native Vanaraja chickens in India.

#### 4.1.4. Agroecology Effect

The lower mean values of most hematological parameters observed in chickens from lowland areas could be due to high environmental temperature, which influences feed and water availability and was similarly reported by Silva [22] and Elnagar et al. [52]. Conversely, the higher RBC levels observed in highland chickens suggest more efficient transporters of O_2_ and CO_2_ through Hb, as previously reported by Chineke et al. [31]. Similarly, the higher MCH value observed in highland chickens indicates an enhanced oxygen‐carrying ability of the blood to carry oxygen, suggesting that birds in high‐altitude environments are more efficient in performing respiratory functions. Aikpitanyi and Egweh [17] noted that increases in the PCV, Hb, and RBC counts of birds indicate an improved oxygen‐carrying capacity of the blood. Moreover, Pendl [46] suggested that a PCV value greater than 56% is an indication of dehydration in most birds. The results of the present study, however, revealed that no dehydration occurred in chickens reared in all agroecological zones, as all PCV values remained within normal physiological ranges.

Regarding the H:L ratio, this parameter is a robust measure of heat stress in birds, as relatively high stress levels generally lead to an increased H:L ratio. A lower H:L ratio in lowlands, particularly during the dry season, reflects the capacity of birds to cope with infection through injury (via heterophil cells) and transmissible disease (via lymphocyte cells) [53]. Minias [18] reported that heterophils form the first line of immune defense against bacterial pathogens in inflammatory lesions, whereas lymphocytes play a key role in humoral adaptive immunity (B cells) and cell‐mediated adaptive immunity (T cells).

### 4.2. Thyroid Hormone Profiles

#### 4.2.1. Breed and Sex Effects

The observed lack of a significant difference between indigenous and Sasso chickens for both T3 and T4 levels might be due to the chickens being evaluated under free‐scavenging conditions. In line with this, Berrong and Washburn [54] reported that more traditional and less selective chicken breeds tend to be more tolerant of both short‐ and long‐term thermal stress than the commercial chickens. This is further supported by Skomorucha and Sosnówka‐Czajka [55], who suggested that chickens with outdoor access possess a normal thermoregulatory system, as evidenced by the reduction in T3 thyroid hormone levels and stable rectal temperature under high daytime temperatures. Furthermore, Sasso chickens are raised under local environmental conditions from hatch and trained to scavenge outdoors, which has favored better adaptation. This can be further explained by the fact that thermal conditioning at an early age results in improved thermotolerance and reduced mortality when re‐exposed to heat stress later in life [56]. Varun et al. [57] also highlighted early thermal conditioning as the most promising method for improving the heat stress adaptability of exotic chickens.

In contrast, the lower, not significantly different, T3 levels observed in indigenous chickens compared to Sasso chickens might be due to their smaller body size. Compared with larger breeds such as Sasso chickens, smaller chickens exhibit lower basal heat production. Similarly, Tixier‐Boichard et al. [58] and Aberra et al. [23] reported that smaller breeds, such as the White Leghorn dwarf strain, have better heat tolerance in stressful environments and lower T3 concentrations. The higher T3 and T4 levels obtained in the present study may be due to the advanced age of the studied chickens, as age and growth rate can influence thyroid hormone concentrations. Arzour‐Lakehal et al. [59] and Bueno et al. [60] reported that T4 concentrations tend to increase with age, whereas T3 levels decrease as chickens mature. Furthermore, in agreement with our observations, Namakparvar et al. [61] reported that male chickens have higher T3 and lower T4 values for the Ross 308, Cobb 500, and Arian breeds.

#### 4.2.2. Season and Agroecology Effects

Season had a significant effect on the hormonal profiles, which was supported by previous studies. For instance, Xie et al. [62] reported a higher mean value of T3 during the cold season (0.92 nmol/L) compared to the warm season (0.84 nmol/L) for RIR chickens. The same study also reported higher T4 values in the cold season (25.7 nmol/L) than in the warm season (19.6 nmol/L), which were higher than the T4 values obtained in our study. Compared with those in warmer environments, elevated T3 and T4 levels were observed in both indigenous and exotic breeds during the cold season, suggesting an increased metabolic rate [63, 64]. This might be due to the thermoregulatory role of T4, as its concentrations rise in response to colder temperatures [65, 66].

The current finding showed that the highland chickens have high levels of both T3 and T4. Similarly, previous studies [67, 68] confirmed that animals living in colder regions tend to have higher T3 and/or T4 levels than those living in warmer regions. Conversely, Tao et al. [69] reported that during the warmer season, chickens exposed to cyclic heat stress exhibited reduced daily mean T3 concentrations and a slower T4 response. Giloh et al. [70] reported a decrease in T3 and an increase in T4 levels in birds exposed to high ambient temperatures during hot weather. The reduction in serum T3 may also result in a response to the reduced thyroid size and secretion rate while increasing T4 concentrations [55].

Moreover, several studies have shown that T3 levels decrease in birds exposed to either induced or environmentally high temperatures, indicating decreased thyroid activity in chickens exposed to heat stress [70–73]; however, T4 levels may remain the same [72] or decrease [52, 70]. Rajkumar et al. [74] reported no significant change in T3 levels between heat‐exposed and unexposed groups. However, Xie et al. [75] reported that birds under acute heat stress showed a decrease in T3 hormone blood levels, whereas those exposed to chronic heat stress showed no change in T3 concentrations. Prolonged high temperature markedly reduces triiodothyronine and thyroxine concentrations in blood serum and plasma [24], which play key roles in metabolic processes. Moreover, thyroid gland size and activity also increase in birds acclimated to low temperatures, suggesting an important role of thyroid hormones in cold acclimation and/or adaptation (MaC Nabb 1985; [65]).

According to a study conducted by Bueno et al. [76], the low levels of T3 and elevated levels of T4 observed in chickens experiencing heat stress were attributed to a reduction in the conversion rate of T4 to T3. This resulted in decreased degradation and an extended half‐life of T4 in the bloodstream. Furthermore, heat‐exposed chickens exhibited significantly lower T3 levels, suggesting decreased thyroid function and a gradual adaptation to prolonged heat exposure [23]. The concentration of T3 in serum is also influenced by dietary carbohydrate levels [77], with a high content of carbohydrates in the diet being associated with an increased concentration of serum T3. In addition, supplementation with iodine in a diet, the inclusion of different levels of rapeseed meal, age, and season significantly influence the levels of T3 and T4 in chicken blood serum [60, 78, 79].

The relatively stable T3 levels observed in this study suggest its potential as an indicator of fitness to heat and cold stresses in both indigenous and Sasso chickens. Thus, the observed hormonal results could serve as a criterion to select chicken breeds resilient to a range of climates, as previously suggested by Bogin et al. [80], Collin et al. [64], and Aberra et al. [73].

## 5. Conclusion and Future Research Plans

A significant variation was observed due to season and agroecology effects, while sex and breed did not exhibit strong significance for most hematological parameters. Despite the absence of significant variations, Sasso chickens showed higher hematological profiles in the highlands and midlands and during wet seasons than in the lowlands and during dry seasons. Furthermore, hormonal analyses showed that no significant variation was observed between indigenous and Sasso chickens, indicating that Sasso chickens had a better adaptive quality under smallholder farmers’ management conditions. Higher T3 and T4 concentrations were observed during the wet season than in the dry season. Likewise, higher T3 and T4 levels were detected in highland, followed by midland chickens. Moreover, the study gave insight into considering seasonal and agroecological influences for susceptible exotic breeds when they are disseminated in various rural farming settings, particularly highlighting the need for tailored management practices that account for these environmental factors. Furthermore, we suggest the use of molecular‐based genotyping, such as SNP‐based markers and RNA‐seq analysis, together with controlled thermal challenge trials to evaluate the real difference between indigenous and Sasso chickens in terms of their climatic resilience potential.

## Author Contributions

Chencha Chebo: writing–review and editing, writing–original draft, visualization, software, resources, methodology, investigation, formal analysis, data curation, conceptualization. Simret Betsha: writing–review and editing, visualization, validation, supervision, conceptualization, and fund administration. Aberra Melesse: writing–review and editing, visualization, validation, supervision, software, formal analysis, conceptualization, fund acquisition. All authors reviewed the manuscript.

## Funding

This study was not supported by any special funding.

## Conflicts of Interest

The authors declare no conflicts of interest.

## Data Availability

Data will be made available on reasonable request from the corresponding author.
